# The COMBINE pneumonia model: a multicenter study to standardize a mouse pneumonia model with *Pseudomonas aeruginosa* and *Klebsiella pneumoniae* for antibiotic development

**DOI:** 10.1128/spectrum.03464-25

**Published:** 2026-01-14

**Authors:** Jon U. Hansen, Rakel Arrazuria, Jennifer L. Hoover, Bernhard Kerscher, Douglas L. Huseby, Sylvie Sordello, Philip Gribbon, Yojana Gadiya, Leonie von Berlin, Gesa Witt, Natália Tassi, Frederikke Rosenborg, Sha Cao, Isabelle Bekeredjian-Ding, Otto Lindahl, Diarmaid Hughes, Lena E. Friberg, Carina Vingsbo Lundberg

**Affiliations:** 1 Department of Bacteria, Parasites and Fungi, Statens Serum Institut 4326, Copenhagen, Denmark; 2 Division of Microbiology, Paul-Ehrlich-Institut 39053, Langen, Germany; 3 Infectious Diseases Research, GSK 525885 , Collegeville, Pennsylvania, USA; 4 Department of Medical Biochemistry and Microbiology, Uppsala University 211745, Uppsala, Sweden; 5 Infectious Diseases, Evotec 165268 , Toulouse, France; 6 Fraunhofer Institute for Translational Medicine and Pharmacology 587462, Hamburg, Germany; 7 Institute of Medical Microbiology and Hospital Hygiene, Marburg University 84932, Marburg, Germany; 8 Department of Pharmacy, Uppsala University 8097, Uppsala, Sweden; Houston Methodist Hospital, Houston, Texas, USA

**Keywords:** Gram-negative, PK/PD, antimicrobial, antimicrobial efficacy studies, lung infection, murine pneumonia model, *K. pneumoniae*, *P. aeruginosa*

## Abstract

The growing threat of antimicrobial resistance highlights the urgent need for new treatment strategies. Reliable animal data are essential to accelerate antibiotic development, and standardized murine infection models, like the neutropenic mouse pneumonia model, can improve the reproducibility and comparability of efficacy data across laboratories—key for clinical translation. This study aims to develop a standardized murine pneumonia model to enhance the clinical relevance of preclinical findings. Using a consensus lung infection protocol, we tested 32 *Klebsiella pneumoniae* and *Pseudomonas aeruginosa* isolates. Fifteen met predefined virulence criteria—showing at least a 1 log_₁₀_ increase in bacterial load from baseline to endpoint, while maintaining mouse survival for at least 12 h post-inoculation. These isolates are available through the German Collection of Microorganisms and Cell Cultures GmbH (DSMZ). Follow-up studies at independent sites confirmed the virulence of eight isolates with minimal variability in bacterial growth. These were added to the Collaboration for Prevention and Treatment of MDR Bacterial Infection (COMBINE Preclinical Bacterial Strain Repository at DSMZ. Based on this work, we propose a standardized experimental framework using this isolate panel to support robust preclinical testing of new antibacterial therapies. This model offers a reproducible, well-characterized platform for evaluating anti-infective candidates. We believe the COMBINE protocol can enhance the reliability and consistency of preclinical efficacy assessments and help reduce the number of animals required, aligning with the 3R principles—reduce, refine, replace—in animal research.

**IMPORTANCE:**

The rise of antibiotic-resistant bacteria has made it increasingly difficult to treat common infections, such as pneumonia. To develop new antibiotics, scientists rely on animal infection models to test how well potential drugs work before human trials. However, inconsistent methods between laboratories make it hard to compare results and slow the progress of new treatments. This study established and validated a standardized mouse pneumonia model for *Klebsiella pneumoniae* and *Pseudomonas aeruginosa*—two major pneumonia pathogens—across three international research centers. By identifying and sharing a set of well-characterized bacterial strains and a common experimental protocol, we provide a reliable foundation for comparing drug efficacy data. This model will help improve the quality and reproducibility of preclinical antibiotic research, reduce unnecessary animal use, and accelerate the discovery of new treatments against life-threatening bacterial infections.

## INTRODUCTION

Antimicrobial resistance (AMR) remains a serious threat to human health, particularly evident in the context of resistant bacterial respiratory infections, which were responsible for more than 400,000 deaths worldwide in 2019 (1). Overall deaths attributed to AMR infections are projected to increase from 4.71 million in 2021 to 8.22 million in 2050, underscoring the urgent need for new therapeutics (2), particularly against Gram-negative species designated by the World Health Organization (WHO) as critical priority pathogens for drug discovery (3). Although promising therapies are under development (4–8), the general picture is that of a thin pipeline dominated by conventional antibiotic classes (9).

Animal infection models play a crucial role in the development and efficacy evaluation of new antimicrobial agents and represent a bridge between *in vitro* preclinical testing and clinical development. A key objective of *in-vivo* efficacy studies is to determine potentially efficacious dosing regimens and pharmacodynamic targets. Moreover, efficacy data from these models are required by regulatory agencies as proof-of-concept prior to clinical trials (10–12). However, progress is hampered by poor reproducibility and a lack of standardized protocols (13, 14).

To address these challenges, the European Innovative Health Initiative (IHI)-funded “Collaboration for Prevention and Treatment of MDR Bacterial Infection” (COMBINE) consortium aims to standardize murine bacterial infection models to enhance the quality and consistency of preclinical efficacy studies.

The choice of animal infection model for efficacy evaluation of novel antimicrobial candidates should be driven by the target clinical indication while also balancing feasibility and ethical considerations. The mouse lung infection model is commonly used for evaluating compounds targeting bacterial species causing pneumonia and, to some extent, mimics the pathophysiological and phenotypic characteristics seen in pneumonia in humans (15, 16). In this study, we focused on *Klebsiella pneumoniae* and *Pseudomonas aeruginosa* as pathogens because of their global significance in the spread of multidrug-resistant strains.

Our team and others have previously highlighted the considerable variability in methodologies used for murine pneumonia models with *K. pneumoniae* and *P. aeruginosa* for testing antimicrobial efficacy of small molecules (13, 17). Key methodological differences expected to influence study outcomes were identified as targets for standardization (18). Rather than attempting to harmonize every parameter—which could limit adoption—we prioritized a minimum set of impactful, feasible parameters for standardization. These include the use of consistently available virulent bacterial isolates; mice of the same sex, breed, immune status, and age; and uniform inoculation techniques. These parameters were extensively discussed with the scientific community, and a consensus protocol was proposed (16).

Here, we present the experimental outcomes from the COMBINE consensus murine lung infection protocol. The selection of bacterial isolates of *K. pneumoniae* and *P. aeruginosa* for this study was guided primarily by the shareability of isolates as one key goal of this effort was to establish a repository of isolates available to the scientific community. Isolate performance in terms of virulence in the consensus pneumonia model was assessed using the following selection criteria: (1) a baseline bacterial load in the lungs of 6–7 log_10_ CFU at 2 h post inoculation; (2) at least one log_10_ increase in the bacterial load from baseline to endpoint; and (3) mice survival for a minimum of 12 h after inoculation. The performance of the isolates was evaluated at 2-3 independent sites, and isolates demonstrating consistent performance across sites are now available to the scientific community via the German Collection of Microorganisms and Cell Cultures GmbH (DSMZ) repository.

## MATERIALS AND METHODS

General conditions as well as standardized parameters of the consensus protocol applicable for all experimental sites are listed in the subsections below and summarized in Table 1. All non-standardized site-specific parameters are listed in supplementary materials.

**TABLE 1:** Standardized parameters and examples of parameters that are not standardized in the protocol for experiments conducted at Statens Serum Institute (SSI), Paul-Ehrlich Institut (PEI) and GSK

	SSI	PEI	GSK
Standardized parameters			
Species, breed, sex, and age	Mouse, CD-1, female, 6–9 weeks when inoculated		
Immune status	Neutropenic by cyclophosphamide: 150 and 100 mg/kg i.p. on day −4 and −1 relative to inoculation respectively.		
Inoculation	Intranasal instillation of 0.05 mL inoculum		
Baseline bacterial burden	6–7 log_10_ CFU in lungs 2 h after inoculation		
Bacterial isolate virulence selection criteria	At least 1 log_10_ CFU increase from baseline to endpoint. At least 12 h before meeting the humane endpoint		
Endpoint	26 h after inoculation (or at humane endpoint)		
Examples of non-standardized parameters			
Specific mouse strain & vendor	Hsd:ICR, Envigo/Inotiv	RjOrl:SWISS, Janvier	Crl:ICR, Charles River
Anesthesia during inoculation	General anesthesia parenteral administration	Inhalation: Isoflurane 3%–5%	
Culture media for inoculum growth	5% horse blood agar plates overnight culture	Trypticase soy broth (log phase after 3-h incubation).	
Culture media for CFU counts	Selective agar	Trypticase soy agar	Blood agar
Sample processing	Stored at −80°C	Freshly processed	

### Bacterial isolates

Initial bacterial *in vivo* screening was performed with clinical isolates kindly provided by NHS Trust Bristol and isolates purchased from ATCC and DSM strain collections. All isolates assigned a DSM ID in Table 2 are now available from DSMZ.

**TABLE 2:** Initial virulence screen in the consensus murine lung infection protocol of 10 *K*. *pneumoniae* and 22 *P*. *aeruginosa* isolates^*a*^

Species	Original isolate ID	Source and year	Inoculum density log_10_ CFU/0.05 mL	Mean log_10_ CFU ± SD in total lung homogenate at 2 h post inoculation	Mean log_10_ CFU ± SD in total lung homogenate at endpoint	Mean Δlog_10_ CFU ± SD in total lung homogenate	Endpoint h post inoculation	Deposited at DSMZ, isolate #
*K. pneumoniae*	ATCC 700603	Urine	6.85	6.21 ± 0.27	4.81 ± 0.88	−1.4 ± 0.88	26	–^*b*^
	KP C1.104	BC, 2019	6.82	6.99 ± 0.17	9.33 ± 0.04	2.34 ± 0.04	26	116098
	KP C1.111	BC, 2019	6.94	7.08 ± 0.12	9.23 ± 0.08	2.15 ± 0.08	26	116097
	KP C1.112	LRT, 2019	6.89	6.97 ± 0.2	9.34 ± 0.02	2.37 ± 0.02	26	116099
	KP C1.113	BC, 2020	6.92	7.04 ± 0.22	8.95 ± 0.21	1.91 ± 0.21	18	116100
	KP C1.147	LRT, 2019	6.88	6.41 ± 0.08	8.36 ± 0.08	1.96 ± 0.08	18	116107
	KP C1.151	BC, 2019	7.24	7.1 ± 0.08	8.42 ± 0.04	1.32 ± 0.04	18.5	116108
	KP C1.181	BC, 2018	6.77	6.71 ± 0.11	8.51 ± 0.04	1.80 ± 0.04	23	116109
	KP C1.184	BC, 2019	6.49	6.46 ± 0.12	5.94 ± 0.57	−0.52±0.57	26	–
	DSM 30104	*Unknown*	6.84	6.28 ± 0.08	8.79 ± 0.42	2.51 ± 0.42	18.5	–
*P. aeruginosa*	ATCC 27853	BC	6.44	5.49 ± 0.05	7.2 ± 0.15	1.70 ± 0.15	10-11	–
	DSM 1128	Ear	6.59	6.12 ± 0.12	6.49 ± 0.36	0.37 ± 0.36	6.5	–
	DSM 50071	*Unknown*	6.75	6.19 ± 0.07	8.39 ± 0.16	2.18 ± 0.16	14.5	–
	DSM 19880 (PA01)	Wound	6.24	5.88 ± 0.1	6.75 ± 0.49	0.88 ± 0.49	11.5	–
	PA 89185	LRT, 2020	6.76	5.44 ± 0.12	6.3 ± 0.06	0.86 ± 0.06	10	–
	PA 89228	BC, 2020	6.51	6.03 ± 0.14	5.44 ± 0.2	−0.59 ± 0.20	16-18	–
	PA 89268	BC, 2020	6.74	5.53 ± 0.17	6.34 ± 0.7	0.81 ± 0.70	18	116110
	PA 89273	LRT, 2020	6.53	5.19 ± 0.36	6.57 ± 0.49	1.37 ± 0.49	10	–
	PA 89337	BC, 2020	6.46	5.81 ± 0.19	5.91 ± 0.3	0.10 ± 0.30	10	–
	PA 89382	BC, 2020	6.86	6.05 ± 0.17	5.95 ± 0.39	−0.09 ± 0.39	10	–
	PA 89399	LRT, 2021	6.64	5.91 ± 0.07	4.81 ± 1.21	−1.09 ± 1.21	10	–
	PA 89580	BC, 2020	6.51	5.85 ± 0.17	6.24 ± 0.12	0.39 ± 0.12	10	–
	PA 88193	BC, 2019	6.65	6.12 ± 0.22	7.16 ± 0.16	1.04 ± 0.16	7.5	–
	PA 88198	BC, 2020	6.88	6.72 ± 0.51	8.14 ± 0.31	1.42 ± 0.31	14	116111
	PA 88245	BC, 2020	6.56	5.97 ± 0.1	6.94 ± 0.18	0.97 ± 0.18	7.5	–
	PA 88276	LRT, 2020	6.93	6.02 ± 0.13	8.12 ± 0.2	2.10 ± 0.20	16	116114
	PA 88337	PFL, 2019	6.81	6.3 ± 0.13	7.47 ± 0.44	1.18 ± 0.44	8	–
	PA 88342	BC, 2020	6.7	6.18 ± 0.14	7.96 ± 0.37	1.78 ± 0.37	12	116115
	PA 88356	BC, 2019	6.95	6.23 ± 0.14	7.99 ± 0.29	1.76 ± 0.29	16	116116
	PA 88361	BC, 2019	6.85	6.15 ± 0.13	8.1 ± 0.47	1.96 ± 0.47	10	–
	PA 88534	LRT, 2019	6.7	6.38 ± 0.16	7.08 ± 0.25	0.70 ± 0.25	8	–
	PA 88826	BC, 2019	6.68	6.12 ± 0.1	7.66 ± 0.3	1.54 ± 0.30	13	116117

^*a*^ Experiments were conducted at SSI. Group size *n *= 5. CFU, colony forming unit; SD, standard deviation. Isolates not meeting the criteria on *in vivo* growth ≥1 log_10_ CFU or endpoint ≥12 h are shaded in gray. BC, blood culture; LRT, lower respiratory tract; PFL, pleural fluid.

^*b*^ The isolates marked with a dash indicate that they were not deposited at DSMZ.

### Animals and housing

Female outbred CD-1 mice 6–9 weeks of age were used for all experiments. Mice were randomized as indicated in supplemental material into groups of 5 and were acclimatized for at least 3 days prior to any experimental procedure. The total number of animals per experiment was between 40 and 80, depending on the number of isolates and time points tested. No mice were excluded during the experiments. Mice were handled by both female and male technicians, and work was blinded as indicated in the supplemental material. The temperature and humidity were registered daily in the animal BSL2 facilities. The mice had free access to domestic quality, autoclaved drinking water and food as specified in the supplemental material. All studies were ethically reviewed and approved by local and national ethical committees and exceeded or met the national animal protection laws, for European contributors based on the European Directive 2010/63/EEC, and for the US contributor on the American Association for the Accreditation of Laboratory Animal Care (AAALAC), the United States Department of Health and Human Services, and all local and federal animal welfare laws.

### Cyclophosphamide

Neutropenia was induced by intraperitoneal (i.p.) injection of cyclophosphamide 150 mg/kg four days prior to inoculation and 100 mg/kg one day prior to inoculation.

### Inoculum

Bacteria in the log phase of growth were suspended in a suitable sterile vehicle at a density that yielded a bacterial load in lungs of 6-7 log_10_ CFU, measured two h after inoculation.

### Inoculation procedure

Under anesthesia, 50 µL of inoculum was deposited in the nares and aspirated while the mouse was positioned in an upright position.

### Monitoring and endpoint

Adhering to national and local rules and guidelines, the clinical status of the mice was monitored throughout the study to identify the time of the humane endpoint and to minimize the suffering of the animals. See supplementary methods for institution-specific endpoints.

### Post mortem and CFU

Bacterial colony counts were determined from lungs sampled at 2–26 h post inoculation. The lungs were removed aseptically, homogenized in a sterile vehicle, and samples were serially diluted and plated on agar media to determine CFU/lungs.

### Statistical analysis

GraphPad Prism 10.0.2 or Excel 2019 was used for data analysis. Delta CFU for each mouse was calculated by subtracting the mean bacterial load at baseline from the mean bacterial load at end point for the respective isolate.

## RESULTS

### Selection of *K. pneumoniae* and *P. aeruginosa* isolates using the consensus protocol

*In vivo* virulence screening was conducted using the consensus pneumonia protocol with 32 *K*. *pneumoniae* and *P. aeruginosa* isolates obtained from ATCC, DSMZ, and clinical sources from NHS Trust Bristol.

Initial screening at a single site (SSI) identified isolates fulfilling the predefined criteria of (i) at least a 1 log_₁₀_ increase in bacterial load from baseline to endpoint and (ii) survival of mice for a minimum of 12 h post-inoculation. Among the *K. pneumoniae* isolates, 8 out of 10 met the bacterial proliferation criteria, demonstrating mild clinical severity with predictable disease progression. Most mice did not reach the humane endpoint within the 26-h experimental window (Table 2).

In contrast, *P. aeruginosa* infections led to more severe and less predictable disease. Fourteen of the 22 isolates tested caused early mortality, with mice reaching humane endpoints before 12 h. Of the eight remaining isolates, one was disqualified due to a decline in mean bacterial burden. One isolate, DSM 116110, showed borderline virulence with high variability (mean increase <1 log_₁₀_) and was retained for further confirmation (Table 2).

A total of 15 isolates—eight *K*. *pneumoniae* and seven *P*. *aeruginosa*—met the selection criteria and were deposited at DSMZ to establish a centralized, quality-controlled isolate repository. All isolates were whole-genome sequenced and subjected to antibiotic susceptibility testing (AST) (Table S1).

### Reproducibility of selected isolates

Five *K. pneumoniae* (DSM 116098, 116097, 116099, 116109, and 30104) and five *P. aeruginosa* isolates (DSM 116110, 116114, 116116, 116117, and 50071) that fulfilled the pre-defined selection criteria in the initial screening were selected for confirmatory studies at SSI, PEI, and/or GSK.

**Fig 1:**
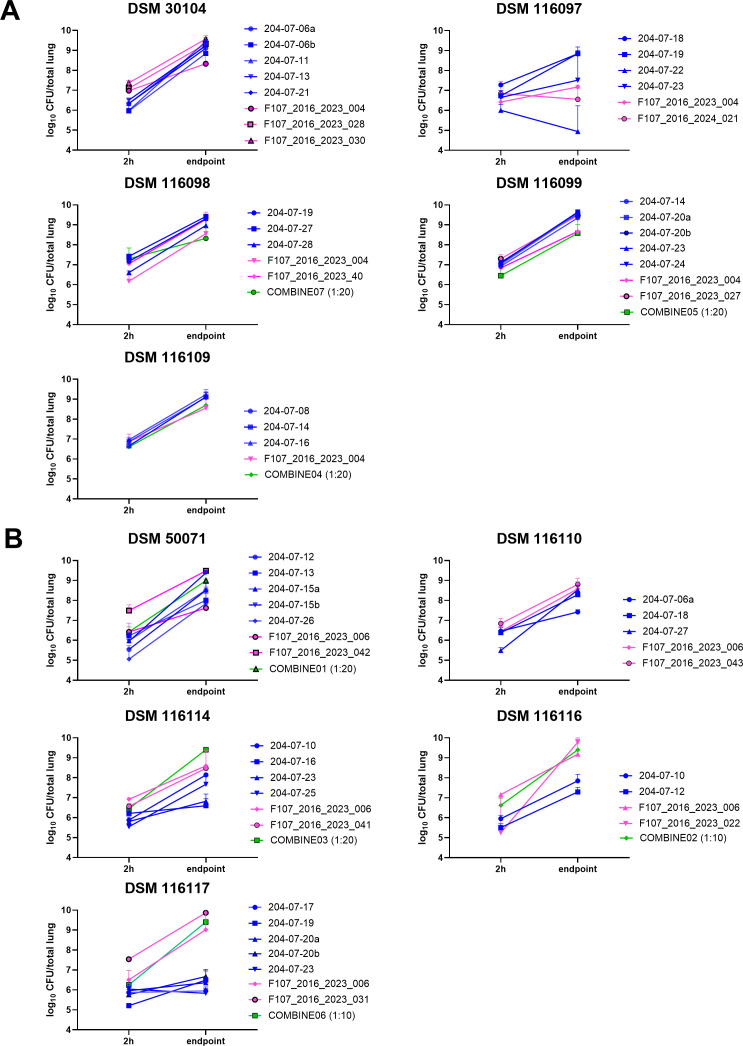
Multicenter confirmation of virulence. Mean + SD bacterial load at baseline and endpoint for the (**A**) five *K. pneumoniae* (DSM 30104, 116097, 116098, 116099, and 116109) and (**B**) five *P. aeruginosa* (DSM 50071, 116110, 116114, 116116, and 116117) isolates selected in the initial screen. The legends indicate the site where the experiment was conducted (Blue = SSI, Magenta = PEI, Green = GSK) and study ID.

**Fig 2:**
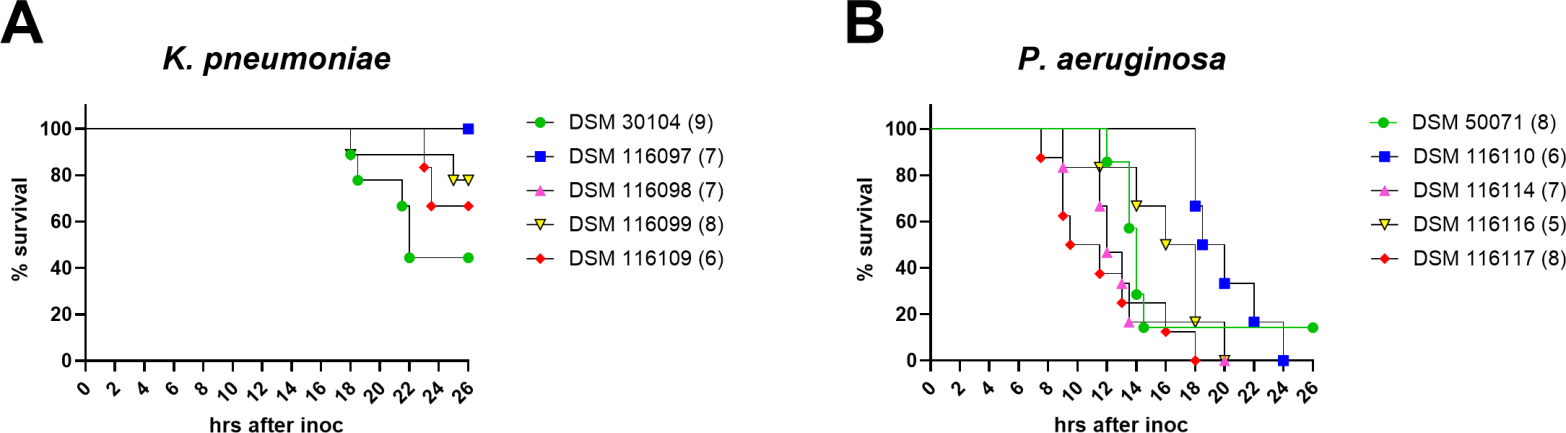
Humane endpoints for selected isolates. Pooled data of same experiments as described in Table 2 and Fig. 1 of (**A**) five *K. pneumoniae* isolates and (**B**) five *P. aeruginosa* isolates. The number in parentheses is the total number of experiments conducted, each with *n* = 5.

Four *K. pneumoniae* isolates (DSM 30104, 116098, 116099, and 116109) showed consistent growth across laboratories (Fig. 1). Time to endpoint varied slightly (Fig. 2), but mice typically survived the full 26-h observation period. An exception was DSM 30104, where 66% of mice (30/45) reached the humane endpoint between 18 and 26 h. DSM 116097 showed inconsistent results across sites and was excluded from further testing.

Among *P. aeruginosa* isolates, DSM 50071, 116110, 116114, and 116116 displayed reproducible *in vivo* performance (Fig. 1). Most experiments showed >1 log_₁₀_ CFU increase, with minor exceptions: one DSM 116110 experiment yielded a mean 0.96 log_₁₀_ increase, and one DSM 116114 experiment showed only a mean 0.39 log_₁₀_ increase. DSM 116117 exhibited inconsistent virulence and was removed from the panel.

The eight consistently well-performing isolates are now part of the COMBINE Preclinical Bacterial Strain Repository (https://www.pei.de/EN/regulation/reference-material/reference-material-node).

### *In vivo* growth kinetics

**Fig 3:**
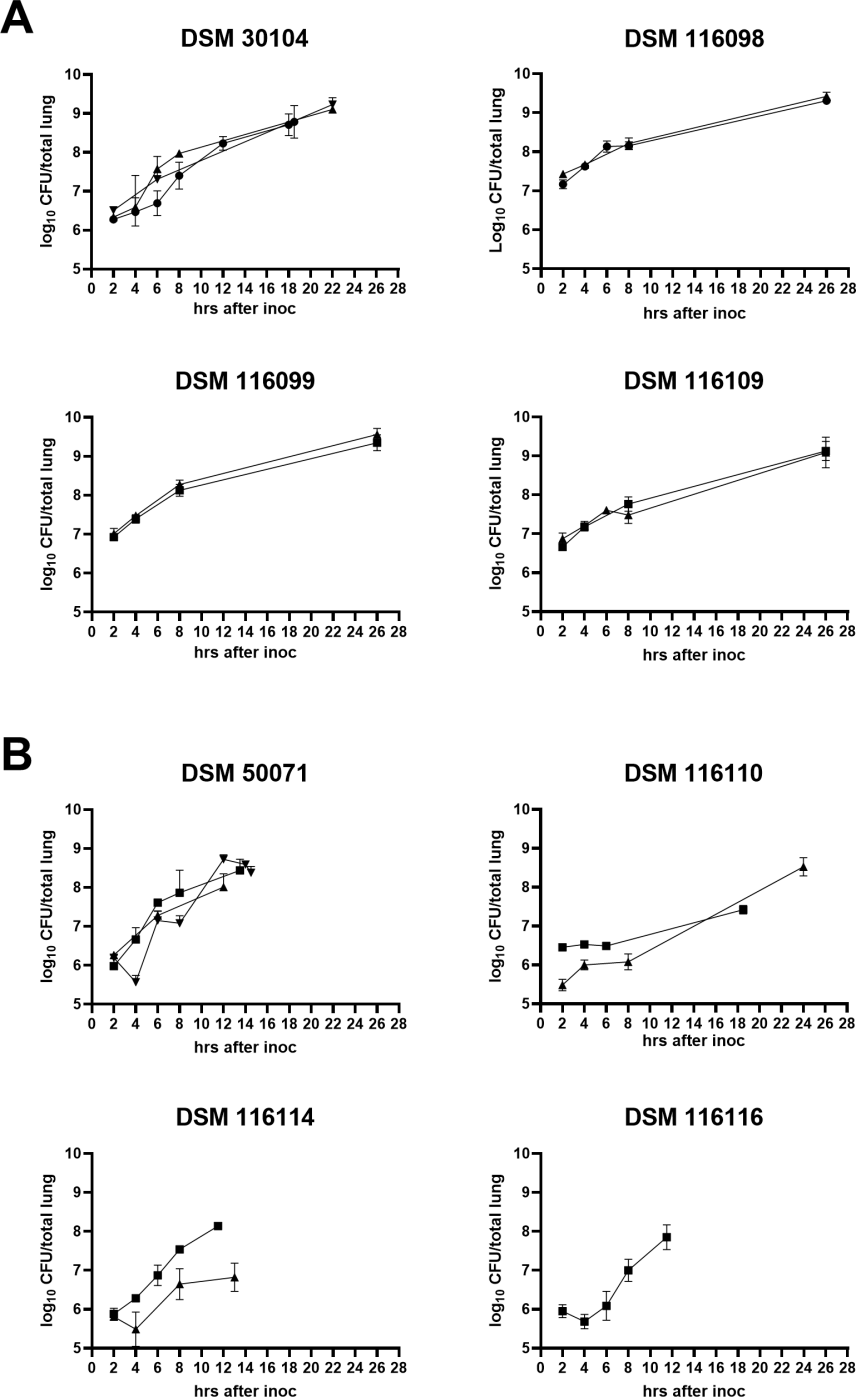
*In vivo* growth curves of selected (**A**) *K. pneumoniae* or (**B**) *P. aeruginosa* isolates in the standard pneumonia model. Experiments were conducted at SSI. Different symbols represent different individual experiments, all with *n* = 5 for each time point.

The kinetics of lung bacterial burden were assessed for the eight isolates that performed consistently across different institutions. *K. pneumoniae* isolates showed uniform *in vivo* growth with no lag phase, increasing by 0.6–1.6 log_₁₀_ CFU between 2 and 8 h post-inoculation and peaking at ~9 log_₁₀_ CFU by 26 h (Fig. 3A). *P. aeruginosa* isolates exhibited more heterogeneous growth kinetics, with some displaying lag phases and inter-experiment variability (Fig. 3B).

Despite slower lung growth for some *P. aeruginosa* isolates, mice often reached the humane endpoint between 10 and 18 h, even when lung burdens remained below 8 log_₁₀_ CFU. All isolates from both species showed bloodstream dissemination at the endpoint, though CFU/mL levels varied widely—from undetectable to >6 log_₁₀_—despite similar lung burdens (data not shown).

### Impact of non-standardized parameters

Although inter-institutional virulence data aligned, experimental conditions were not identical (Table 1) and could potentially result in variability between laboratories. We looked into the impact of two of the non-standardized parameters that differed in the protocols of the participating laboratories: (i) the anesthesia used during the inoculation and (ii) the culture conditions of the bacteria used for preparing the inoculum. To understand the potential impact of these differences, SSI performed studies comparing these experimental settings within the same study. No difference in growth rate, endpoint CFU, or time to endpoint was observed for *K. pneumoniae* DSM 30104 when comparing the two different applications of anesthesia during the inoculation (Fig. S2A). When comparing the impact of the different culture conditions for preparing the inoculum, *K. pneumoniae* DSM 116099 displayed almost identical growth rates, endpoint CFUs, and time-to-endpoint regardless of the pre-experimental culture conditions (Fig. S2B). In contrast, when comparing culture conditions for *P. aeruginosa* DSM 50071, a clear difference was observed with the inoculum prepared from log-phase broth culture having a lag phase and delayed endpoint (Fig. S2C) when compared with the overnight agar culture.

### Additional isolate characterization

To support model reproducibility and utility, the 15 isolates identified in Table 2 were whole-genome sequenced, and reference genomes uploaded to the NCBI database. Sequence types, genome lengths, plasmid content, serotype, resistance genes, and MIC profiles are detailed in Fig. S1 and Table S1

### Compliance with FAIR principles

Our goal in the COMBINE project is to provide sustainable solutions that enable researchers to apply or expand our consensus pneumonia model and compare results across studies. To achieve this, we not only deposited the selected isolates into DSMZ but also ensured the compliance of the accompanying data with the Findable, Accessible, Interoperable, and Reusable (FAIR) principles (19). When fully implemented, the FAIR principles enhance data discoverability, usability, and integration across studies, ultimately improving reproducibility and facilitating collaboration (20, 21).

To support these principles, we created structured machine-readable data templates to standardize reporting, ensuring that key experimental details are consistently documented. For some variables such as experiment type, biomaterial, culture media, statistical methods, and result units, we used controlled vocabularies from published ontologies making the data interoperable. The FAIRness of the structured data template was evaluated using the FAIR Data Set Maturity (DSM) model (https://fairplus.github.io/Data-Maturity/) in order to govern compliance with the FAIR principles. In addition, we deposited the data and metadata on Zenodo (https://zenodo.org/records/15124939) to ensure its accessibility through a Digital Object Identifier (DOI) and long-term, searchable storage. These measures collectively improve the transparency, reliability, and reusability of the data, enabling other researchers to build upon our findings.

## DISCUSSION

The present study aimed to assess the outcome of the consensus protocol proposed by the COMBINE consortium (18) and to establish a standardized panel of virulent *K. pneumoniae* and *P. aeruginosa* isolates for use in this model. By doing so, we sought to develop a reliable preclinical pneumonia model for evaluating novel antimicrobial compounds. The isolates that were identified to fulfill the pre-defined virulence criteria are now available at the DSMZ repository, and all data and meta-data from experiments presented in this manuscript are accessible at Zenodo according to the FAIR principles.

The reproducibility of the virulence characteristics was confirmed across multiple independent laboratories in different countries: Statens Serum Institut in Denmark, Paul-Ehrlich-Institut in Germany, and GSK in the United States. Additionally, seven out of the eight isolates presented in Fig. 3 were also found to proliferate *in vivo* in a recent independent study in a separate lab (22), further validating the robustness and consistency of the COMBINE standard protocol. This strengthens the premise that a standardized, well-characterized model enhances the comparability of results across different research settings.

Adopting validated isolates and harmonized procedures reduces the need to develop new models from scratch, thus supporting the 3Rs principle (Replacement, Reduction, Refinement). Importantly, the protocol’s reproducibility minimizes inter-laboratory variability and helps decrease overall animal usage. Given the significant methodological inconsistencies in current literature on murine pneumonia models (13), standardization and transparent reporting—aligned with the ARRIVE guidelines (23)—are essential for reproducible and ethically responsible research.

The choice of bacterial isolate significantly affects the pneumonia model phenotype. Within the COMBINE project, we prioritized isolates that consistently proliferated in murine lungs and induced measurable disease without causing mortality within 12 h—criteria suited for evaluating small-molecule antibiotics. However, this may introduce a bias, particularly for *P. aeruginosa*, where several virulent isolates were excluded due to rapid clinical deterioration.

Ensuring accurate quantification of the inoculum size and maintaining consistency across independent experiments is critical when testing antibacterial compounds in animal infection models (24). In our studies, all inoculum suspensions were prepared based on an initial optical density measurement, in general aiming for 6.7 log_10_ CFU/0.05 mL in order to reach a relatively high baseline burden in lungs of 6–7 log_10_ CFU, typically recommended for small-molecule antibiotic evaluations (25). While the inoculum preparations used for different experiments with the same isolate for the most part varied no more than 0.3 log_10_ CFU (data not shown), the baseline burden in lungs did vary more than that, also for experiments conducted at the same site (Fig. 1). We investigated a few variables that could potentially influence baseline burden and bacterial growth and compared two different culturing methods for inoculum preparation: agar culture versus log-phase broth culture, and choice of anesthesia; parenteral administration of long-lasting anesthesia or inhaled short-duration anesthesia. For the inoculum growth media, we observed that while the final bacterial loads were comparable, depending on the strain tested, the *in vivo* growth rate appeared to be influenced by the culture conditions. Further studies are required to determine whether these observations hold true for additional bacterial isolates. Different anesthesia protocols were also employed for the intranasal inoculation across laboratories—either parenteral general anesthesia lasting h or inhaled general anesthesia lasting only a few minutes. Choice of anesthesia has been demonstrated to affect delivery into the lungs and infection outcome (26), and although we observed no impact on bacterial growth, we cannot exclude that other bacterial isolates could be affected by the anesthesia administration method.

To accurately evaluate disease progression, a non-invasive and detailed monitoring of the clinical status and well-being of the mice is required. Human observations following a scoring of clinical symptoms of discomfort are useful methods to define humane endpoints and are employed as a surrogate marker for death in survival studies with the intention to minimize the suffering of the animals and to adhere to the 3R principles. Especially for *P. aeruginosa* infections, our study highlights the relatively high variability in time to humane endpoint between experiments. Further refinement, e.g., automated tracking of activity levels and body temperature could provide an earlier indication of disease manifestation and possibly enable a less variable scoring of disease course and severity.

While the standard pneumonia model presented here is developed to be suitable for antimicrobial efficacy characterization of small molecules against *K. pneumoniae* and *P. aeruginosa* in 24-h PKPD studies, it may or may not be suitable for other bacterial species, other drug modalities, or other durations of the study period, such as in chronic infection models. Currently, additional studies are ongoing to ascertain whether this standardized protocol, with some modifications, is applicable to other clinically relevant bacterial species, such as *Acinetobacter baumannii* and methicillin-resistant *Staphylococcus aureus* (MRSA) (27)*,* where there is also a need for a harmonized and validated model for evaluation of novel antibiotic treatment options.

In conclusion, we present a standardized murine pneumonia model, along with a panel of well-characterized *K. pneumoniae* and *P. aeruginosa* isolates, to the research community. These isolates demonstrate consistent virulence with low variability, providing a robust foundation for evaluating antimicrobial efficacy. A reproducible model is a prerequisite for generating high-quality efficacy data and translating preclinical findings into clinical success. Ongoing work within the COMBINE consortium aims to further define PK/PD parameters for key antibiotics using this model, thereby advancing drug development against multidrug-resistant respiratory pathogens.

## Data Availability

All the data generated in this study have been deposited across domain-specific repositories (if available) for re-use by the community. The isolates are deposited on the DSMZ repository (https://www.dsmz.de/) for replication studies with DSM IDs as indicated in Table 2. The experimental results can be found on Zenodo (https://zenodo.org/records/15124939) together with the research protocol to build machine ready datasets. Annotated genomes were deposited the in the NCBI Genome database with the Bioproject accession number PRJNA1208266.

## SUPPLEMENTAL MATERIAL

Figure S1 spectrum.03464-25-s0001.pdf Genome sequenced strains.

Figure S2 spectrum.03464-25-s0002.pdf Non-standardized parameters.

Supplemental Materials and Methods spectrum.03464-25-s0003.docx Site-specific variables.

Table S1 spectrum.03464-25-s0004.pdf Isolate genotype and AST.

ASM does not own the copyrights to Supplemental Material that may be linked to, or accessed through, an article. The authors have granted ASM a non-exclusive, world-wide license to publish the Supplemental Material files. Please contact the corresponding author directly for reuse.
