# Supplementary figures and images for: The COMBINE pneumonia model: a multicenter study to standardize a mouse pneumonia model with Pseudomonas aeruginosa and Klebsiella pneumoniae for antibiotic development
Source: Microbiol Spectr. 2026 Jan 14;14(3):e03464-25. doi: 10.1128/spectrum.03464-25 (PMC12955464; doi:10.1128/spectrum.03464-25)

**Figure S1.** Genomic architecture, sequence type and serotype of sequenced isolates.

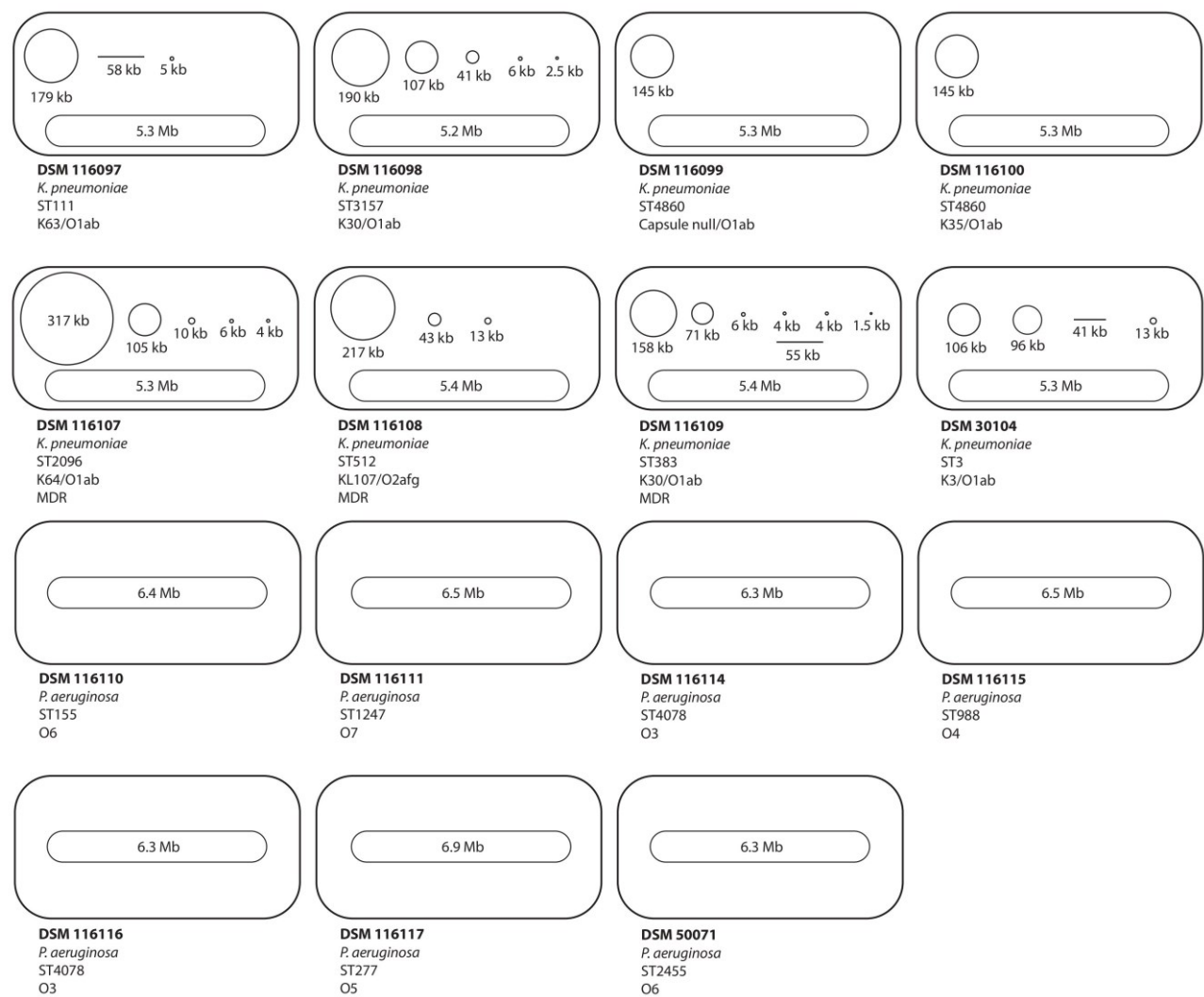

Supplement: Figure S1 — Genome sequenced strains. [file spectrum.03464-25-s0001.pdf]
