# Supplementary material for: The COMBINE pneumonia model: a multicenter study to standardize a mouse pneumonia model with Pseudomonas aeruginosa and Klebsiella pneumoniae for antibiotic development
Source: Microbiol Spectr. 2026 Jan 14;14(3):e03464-25. doi: 10.1128/spectrum.03464-25 (PMC12955464; doi:10.1128/spectrum.03464-25)

Figure S2 Effect of difference in anesthesia or inoculum growth media on *in-vivo* growth. A) Mean±SD bacterial burden in lungs of mice inoculated intranasally with *K. pneumoniae* DSM 30104 following different anesthesia protocols: General anesthesia as typically used at SSI or isoflurane anesthesia as typically used at PEI and GSK. B) Mean±SD bacterial burden in lungs of mice inoculated intranasally with *K. pneumoniae* DSM 116099 and C) *P. aeruginosa* DSM 50071 inoculum prepared from over-night agar culture typically used at SSI or log-phase broth culture typically used at PEI and GSK.

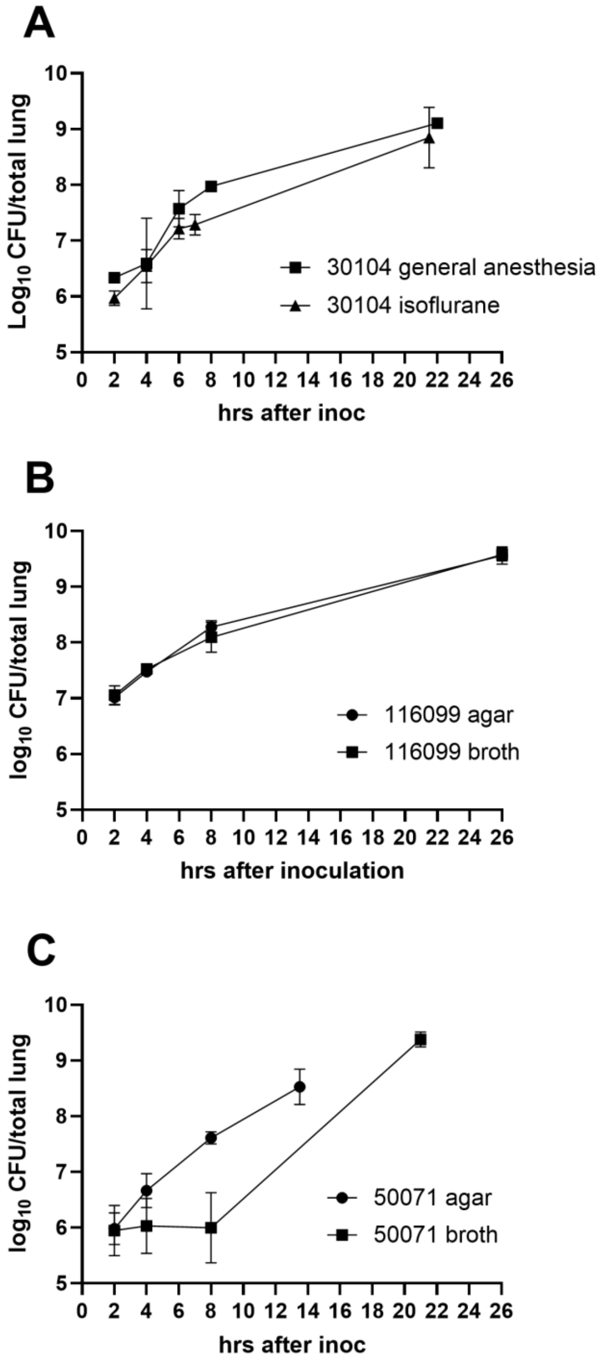

Supplement: Figure S2 — Non-standardized parameters. [file spectrum.03464-25-s0002.pdf]
