# Supplementary material for: The COMBINE pneumonia model: a multicenter study to standardize a mouse pneumonia model with Pseudomonas aeruginosa and Klebsiella pneumoniae for antibiotic development
Source: Microbiol Spectr. 2026 Jan 14;14(3):e03464-25. doi: 10.1128/spectrum.03464-25 (PMC12955464; doi:10.1128/spectrum.03464-25)
Supplement: Supplemental Materials and Methods — Site-specific variables. [file spectrum.03464-25-s0003.docx]

## Supplemental Material & Methods

### Animals & Housing

**SSI specific parameters**: Hsd:ICR (CD-1) female mice from Envigo/Inotiv (NL), 5-6 weeks old on arrival, 7-8 weeks old when inoculated. Randomized by pick order into groups of 5. Acclimatization period 9 days. Approx. 30-gram body weight at experiment start. Feed: Teklad Global diet 2916C-Envigo and occasionally peanuts, sunflower seeds and corn kernels (Irradiated, Brogaarden). Housing : Type 3 macrolone cages with bedding from Tapvei and Tapvei S-brick Aspen and Diamond twists enrichment. Further, the animals were offered Enviro-Dri nesting material and cardboard houses (Bio-serv). Cages were changed once weekly and also 24 h after last cyclophosphamide dose, re-using enrichments and unspoiled bedding material. Danish license No. 2021-15-0201-01055

**PEI specific parameters**: RjOrl:SWISS (CD-1) female mice from Janvier (FR), 6-8 weeks old at the start of any intervention. Feed: Rat/mouse maintenance, 10 mm sterilized 25 kGy V1534-727 from Ssniff). Housing: techniplast blue line cages with NestPak Aspen Grade 6 bedding (Datesand) without additional enrichment. Cages were changed once weekly until the first cyclophosphamide dose. Regierungspraesidium of the State of Hesse project license No. F1072016).

**GSK specific parameters**: Crl: ICR(CD-1) female mice from Charles River (Kingston, NY) were 6-8 weeks old on arrival and 7-9 weeks old and approximately 30g at study start. Animals were housed in groups of five in IVC Innoviv boxes on alpha dry bedding with standard 12-hour light/dark cycles, room temperature of 68 to 79 °F and humidity of 30% - 70%. They were allowed access to Lab DIET-Rodent diet 5001 food and sterile filtered bottled water ad libitum and provided with nestlets and huts for enrichment. Animals were randomized by body weight at the time of arrival using a proprietary GSK statistical application. All studies were conducted according to GSK's Policy on the Care, Welfare and Treatment of Animals and reviewed by the Institutional Animal Care and Use Committee at GSK.

### Cyclophosphamide

**SSI specific parameters**: Injection volume 0.5 mL per mouse. Product: Sendoxan, Baxter. Doses were based on average body weight of all mice in the experiment measured on day -5.

**PEI specific parameters**: Injection volume 10 mL/kg, Product: Endoxan, Baxter. Doses were adjusted to individual animal weight on the day of dosing.

**GSK specific parameters**: Cyclophosphamide as a 20 mg/mL solution was obtained from Hanna Pharmaceuticals, diluted with saline to 15 mg/mL (day -4) and 10 mg/mL (day -1), and injected at a volume of 0.3 mL per mouse based on average body weight of 30g.

### Inoculum

**SSI specific parameters**: Bacteria were grown overnight (14-18 h) on 5% horse blood agar (SSI Diagnostica) at 35°C ambient air and single colonies were suspended in sterile 0.9% NaCl to an OD_546_=0.13, corresponding to approximately 2x10^8^ CFU/mL. Alternatively, TSB broth was prepared according to manufacturer instructions, with 30 grams dissolved in 1 litre WFI and autoclaved 121°C for 15 min. 10 µl bacteria from cryo-stock was seeded in 1.5 ml TSB and 1 ml was then transferred to 50 ml TSB in a 250 ml flask and incubated in ambient air at 36ºC with shaking overnight. After 14-18 h, 1 mL was transferred into 50 mL fresh TSB broth and incubated for 3h at 36ºC with shaking. The bacteria were harvested by centrifugation for 5 minutes at 4000 RCF and 20°C. The pellet was washed three times by resuspension in 10 mL 0.9% NaCl and centrifugation as described above. The inoculum was prepared by adding 4-9 drops of bacterial suspension to 5 mL 0.9% sterile NaCl to reach OD_546_=0.13 For all isolates, prior to animal experiments the bacterial density at OD_540_=0.13 was verified and dilution factors calculated to reach an inoculum size of 2x10^8^ CFU/ml for *K. pneumoniae* and 1x10^8^ CFU/ml for *P. aeruginosa*, corresponding to 10^7^ and 5x10^6^ CFU instilled via the nares, respectively. The inoculum was prepared 45-90 minutes prior to inoculation, maintained at RT until administered, and quantified by 10-fold serial dilution in 0.9% NaCl and application of 20 μL spots to bromothymol “blue” agar plates (SSI Diagnostica) for *K. pneumoniae* or Cetrimide agar plates (SSI Diagnostica) for *P. aeruginosa*.

**PEI specific parameters:** Log-phase cultures were obtained by inoculation of 25 mL tryptic soy broth with an overnight culture from frozen stocks at a dilution of at least 1:400, followed by incubation at 37°C in ambient air with agitation at 200 rpm for approximately 3 h until an OD_600_ of 0.8-1 was reached. Bacteria were harvested by centrifugation (5000 x g, 5 min at 15°C), washed twice with PBS and adjusted to a density of 2x10^8^ CFU/ml in PBS, resulting in 6-7 log_10_ CFU in lungs 2 h after inoculation. The final inoculum was quantified by plating serial dilutions on tryptic soy agar to confirm correct inoculum concentrations.

**GSK specific parameters:** Frozen stocks were thawed and subcultured by adding 100 uL to approximately 50 mL TSB (Becton Dickinson) and incubating overnight at 37 C in ambient air with gentle agitation (approx. 125 rpm). The day of infection, a log phase culture was created by adding 1 mL of the overnight culture to 50 mL fresh TSB and incubating as indicated above for 3 h. Bacteria were harvested by centrifugation for 5 minutes at 4200 RCF at room temperature, resuspended in 35 mL sterile saline (0.9% NaCl) and centrifuged again. This was repeated two more times, and the final bacterial pellet was resuspended in 5 mL sterile saline. The final resuspension was further adjusted using sterile saline to a pre-determined OD600 corresponding to approximately 8-9 log_10_ CFU/ml. The final inoculum was quantified by plating 20 uL triplicate aliquots of 10-fold serial dilutions on trypticase soy agar plates supplemented with 5% sheep blood (Hardy Diagnostics) and incubating overnight prior to assessment.

### Inoc**ulation pr**ocedure

**SSI specific parameters:** Mice were anesthetized with 0.15 ml s.c. of Zoletil general anesthesia (Zolazepam 15 mg/kg, Tiletamin 15 mg/kg, Narcozyl 24 mg/kg, Torbugesic 0.3 mg/kg) and in an upright position 0.05 ml of the bacteria suspension was slowly deposited at the nares with a pipette and aspirated. The mice rested in a supine position with their heads slightly elevated at 35°C until fully awake from anesthesia, approx. 4-5 h after inoculation, Alternatively, anesthesia was induced by isoflurane (Vetflurane) inhalation (Oxygen flow 2 L/min, 2-3 % isoflurane) and a 50 µL inoculum volume was instilled via the nares and inhaled while held in an upright position. The mice recovered within minutes. Mice were housed at room temperature for the remainder of the experiment.

**PEI specific parameters**: Mice were anaesthetized by inhalation of 3-5 % isoflurane (Isofluran Baxter vet., 1000 mg/g) vaporized at a flow rate of 0.8 L/min (indulab-vet) in oxygenated air (JAY-5, Longfian Scitech), manually restrained (held upright) and infected intranasally by deposition of 50 μl of bacterial suspension to the nares and allowing the mice to inhale the inoculum. After the inoculum was inhaled, mice were placed back into their cages and recovered generally within one minute. Mice were housed at room temperature for the remainder of the experiment.

**GSK specific parameters**: Animals were anesthetized by exposure to inhaled isoflurane (3-5% in 1.5 L/min oxygen) for approximately 4 minutes until muscle tone was fully relaxed and no reflex was observed upon toe pinch. The bacterial suspension was kept at room temperature while the mice were infected. The mice were infected intra-nasally by placing 50 µL of bacterial suspension on the nares and allowing the mice to inhale the inoculum. Animals were returned to their home cage and observed until recovered from anesthesia.

### Monitoring & Endpoint

**SSI specific parameters:** The mice were observed for clinical signs every 2-6 h after inoculation using a scoring system based on grimace scale and activity level to assess disease progression. Upon reaching a moderate clinical score defined by light piloerection, pinched eyes and reduced or slower movements, mice received buprenorphine (0.07 mg/kg) analgesia and were evaluated more frequently at 1-2 h intervals. Mice were euthanized after 6 h at a moderate score and prior to progressing to a severe score (piloerection, half closed, stationary).

**PEI specific parameters**: Mice were monitored regularly depending on the progression of the infection and their health status scored until the humane or experimental endpoint was reached.

**GSK specific parameters**: Mice were monitored at 5- and 15-minutes post infection and regularly at least twice daily for humane endpoints. Animals were euthanized upon reaching the humane endpoints or at the end of the study period (26h post infection).

### Post mortem & CFU

**SSI specific parameters**: The mice were sacrificed by cervical dislocation, and lungs were excised aseptically and stored at -80°C until the experiment completed. All study samples were thawed and processed at the same time. Upon thawing, the lungs were homogenized in 2 ml PBS using two 2 mm sterile steel beads and a Qiagen tissuelyser II running at 30 Hz for 2 minutes. The homogenate was serially diluted 10-fold in PBS. Then, 20 μL spots were applied in duplicate on blue agar plates (SSI Diagnostica) for *K. pneumoniae* isolates and cetrimide agar plates (Thermo scientific) for *P. aeruginosa* inoculated animals. Agar plates were incubated 18-22 h at 35°C in ambient air prior to assessment of colony counts. Colonies were counted from the first dilution yielding a ‘quantifiable’ number (e.g., approximately 10-50 colonies per aliquoted spot).

**PEI specific parameters:** At this point or if humane endpoints were reached earlier, animals were euthanized by CO_2_ overdose and cervical dislocation. Lungs were excised aseptically and collected in FastPrep Lysematrix D tubes (MP Biomedicals) with 1 mL PBS, 0.1 % Triton X-100, and processed at the time of collection. Lungs were disintegrated with 3 cycles of bead-beating (5000rpm, 13 seconds/cycle with 15 second breaks) on a FastPrep-24 (MP Biomedicals). Homogenates were serially diluted in PBS and 20 µL aliquots plated on tryptic soy agar in duplicates and plates incubated overnight at 37°C prior to assessment of colony counts. Colonies were counted from the first dilution yielding a ‘quantifiable’ number (e.g., approximately 10-50 colonies per aliquoted spot).

**GSK specific parameters**: Mice were euthanized with controlled rising levels of CO_2_, followed by cervical dislocation to confirm death. Lungs were excised aseptically and placed into sterile processing bags with 1 mL sterile saline. Sample were homogenized for 2 minutes using a laboratory blender (Seward stomacher). Homogenates were serially diluted 10-fold in sterile saline and 20 uL aliquots were plated in triplicate onto blood agar plates using a liquid handling system (Hamilton). Agar plates were blinded by covering the sample identifier with an opaque sticker and incubated approximately 24 h at 37°C in ambient air prior to assessment of colony counts. Colonies were counted from the first dilution yielding a ‘quantifiable’ number (e.g., approximately 10-50 colonies per aliquoted spot) by an independent scientist not associated with the study; after recording the colony counts, results were provided to the study investigator for unblinding and analysis.

### Whole genome sequencing of the 15 isolates deposited at DSMZ

Genomic DNA was extracted using a MasterPure Complete DNA & RNA Purification Kit (Lucigen). DNA was sequenced using both Illumina short-read and Oxford Nanopore long-read technologies. For Illumina sequencing, genomic libraries were prepared using a Nextera XT library preparation kit followed by sequencing using a MiSeq device with a V3 600-cycle reagent kit. For Nanopore sequencing, genomic libraries were prepared using a Rapid Barcoding Kit V14 followed by sequencing on a MinION Mk1C device using a R10 flow cell.

The long-read and short-read data were *de novo* assembled into reference genomes using either Unicycler (28) or Trycycler (29). Assemblies were checked for completeness and accuracy and corrected using CLC Genomics Workbench (Qiagen). The resulting completed sequences were then annotated using the NCBI prokaryote genomic annotation pipeline (30). Antibiotic resistance genes in all genomes were identified using the ResFinder database (31, 32). *Klebsiella pneumoniae* genomes were further analyzed using Kleborate (33, 34) to identify serotypes, sequence type and OMP mutations. *Pseudomonas aeruginosa* isolates were serotyped using Past (35) and the sequence type was determined using MLST 2.0 (36).

Annotated genomes were deposited the in the NCBI Genome database with the Bioproject accession number PRJNA1208266

### Antibiotic susceptibility

The antibiotic susceptibility of isolates was determined by broth microdilution according to Clinical and Laboratory Standards Institute guidelines.

### References

28. Wick RR, Judd LM, Gorrie CL, Holt KE. 2017. Unicycler: resolving bacterial genome assemblies from short and long sequencing reads. PLoS Comput Biol 13:e1005595. <https://doi.org/10.1371/journal.pcbi.1005595>

29. Wick RR, Judd LM, Cerdeira LT, Hawkey J, Méric G, Vezina B, Wyres KL, Holt KE. 2021. Trycycler: consensus long-read assemblies for bacterial genomes. Genome Biol 22:266. <https://doi.org/10.1186/s13059-021-02483-z>

30. Li W, O’Neill KR, Haft DH, DiCuccio M, Chetvernin V, Badretdin A, Coulouris G, Chitsaz F, Derbyshire MK, Durkin AS, Gonzales NR, Gwadz M, Lanczycki CJ, Song JS, Thanki N, Wang J, Yamashita RA, Yang M, Zheng C, Marchler-Bauer A, Thibaud-Nissen F. 2021. RefSeq: expanding the prokaryotic genome annotation pipeline reach with protein family model curation. Nucleic Acids Res 49:D1020–D1028. <https://doi.org/10.1093/nar/gkaa1105>

31. Bortolaia V, Kaas RS, Ruppe E, Roberts MC, Schwarz S, Cattoir V, Philippon A, Allesoe RL, Rebelo AR, Florensa AF, et al. 2020. ResFinder 4.0 for predictions of phenotypes from genotypes. J Antimicrob Chemother 75:3491–3500. <https://doi.org/10.1093/jac/dkaa345>

32. Camacho C, Coulouris G, Avagyan V, Ma N, Papadopoulos J, Bealer K, Madden TL. 2009. BLAST+: architecture and applications. BMC Bioinformatics 10:1–9. <https://doi.org/10.1186/1471-2105-10-421>

33. Lam MMC, Wick RR, Watts SC, Cerdeira LT, Wyres KL, Holt KE. 2021. A genomic surveillance framework and genotyping tool for *Klebsiella pneumoniae* and its related species complex. Nat Commun 12:4188. <https://doi.org/10.1038/s41467-021-24448-3>

34. Wyres KL, Wick RR, Gorrie C, Jenney A, Follador R, Thomson NR, Holt KE. 2016. Identification of *Klebsiella* capsule synthesis loci from whole genome data. Microb Genom 2:e000102. <https://doi.org/10.1099/mgen.0.000102>

35. Thrane SW, Taylor VL, Lund O, Lam JS, Jelsbak L. 2016. Application of whole-genome sequencing data for O-specific antigen analysis and *in silico* serotyping of *Pseudomonas aeruginosa* isolates. J Clin Microbiol 54:1782–1788. <https://doi.org/10.1128/JCM.00349-16>

36. Larsen MV, Cosentino S, Rasmussen S, Friis C, Hasman H, Marvig RL, Jelsbak L, Sicheritz-Pontén T, Ussery DW, Aarestrup FM, Lund O. 2012. Multilocus sequence typing of total-genome-sequenced bacteria. J Clin Microbiol 50:1355–1361. <https://doi.org/10.1128/JCM.06094-11>
