# Supplementary material for: The COMBINE pneumonia model: a multicenter study to standardize a mouse pneumonia model with Pseudomonas aeruginosa and Klebsiella pneumoniae for antibiotic development
Source: Microbiol Spectr. 2026 Jan 14;14(3):e03464-25. doi: 10.1128/spectrum.03464-25 (PMC12955464; doi:10.1128/spectrum.03464-25)
Supplement: Table S1 — Isolate genotype and AST. [file spectrum.03464-25-s0004.pdf]

Table S1, Antibiotic resistance characteristics and genotypes of isolates. Kpn, *K. pneumoniae*. Pae, *P. aeruginosa*. MEM, meropenem. LFX, levofloxacin. GEN, gentamicin. CAZ, ceftazidime

Isolate DSM #	Species	Intrinsic Resistance Genes	Acquired Resistance Genes	Minimum inhibitory concentration (mg/L)			
				**MEM**	**LFX**	**GEN**	**CAZ**
116097	*Kpn*	blaSHV, fosA6, OqxA, OqxB		0.032	0.125	1	0.5
116098	*Kpn*	blaSHV, fosA6, OqxA, OqxB		0.032	0.0625	1	0.25
116099	*Kpn*	blaSHV, fosA6, OqxA, OqxB		0.032	0.125	0.5	0.25
116100	*Kpn*	blaSHV, fosA6, OqxA, OqxB		0.032	0.125	1	0.25
116107	*Kpn*	blaSHV, fosA6, OqxA, OqxB	aac(6')-Ib-cr, aadA2, armA, blaOXA-1, blaCTX-M-15, blaOXA-232, blaTEM-1A, mph(E), msr(E), catB3, sul1, tet(D), dfrA1, dfrA12, dfrA14, *ompK36GD*	8	32	>64	>64
116108	*Kpn*	blaSHV, fosA6, OqxA, OqxB	aadA2, aac(6')-Ib, blaKPC-3, mph(A), catA1, sul1, dfrA12, *ompK36GD*, *ompK35* truncation	64	64	2	>64
116109	*Kpn*	blaSHV, fosA6, OqxA, OqxB	aph(3')-Ia, aadA2, aph(3'')-Ib, aph(6)-Id, aac(6')-Il, aadA1, aac(3)-IIa, blaVIM-19, blaCMY-4, mph(A), sul1, sul2, tet(A), dfrA12, dfrA1	16	32	64	>64
30104	*Kpn*	blaSHV, fosA6, OqxA, OqxB		0.032	0.125	0.25	0.25
116110	*Pae*	aph(3')-IIb, blaOXA-396, blaPAO (PDC-5), fosA, catB7		0.5	8	1	2
116111	*Pae*	aph(3')-IIb, blaOXA-494, blaPAO (PDC-3), fosA, catB7		0.25	8	1	4
116114	*Pae*	aph(3')-IIb, blaOXA-1125, blaPAO (PDC-5), fosA, catB7		0.125	0.5	2	32
116115	*Pae*	aph(3')-IIb, blaOXA-904, blaPAO (PDC-23), fosA, catB7		0.0625	0.5	2	2
116116	*Pae*	aph(3')-IIb, blaOXA-1125, blaPAO (PDC-5), fosA, catB7	*oprD* interruption (fs at nt 413)	8	4	8	>64
116117	*Pae*	aph(3')-Iib, blaOXA-494, blaPAO (PDC-5), fosA, catB7		0.25	0.5	2	2
50071	*Pae*	aph(3')-IIb, blaOXA-904, blaPAO (PDC-5), fosA, catB7		1	2	8	4
ATCC 25922	*E. coli*	-	-	0.032	0.016	1	0.5
